# All-Fiber Highly Sensitive Bragg Grating Bend Sensor

**DOI:** 10.3390/s19194228

**Published:** 2019-09-28

**Authors:** Oleg V. Butov, Alexey P. Bazakutsa, Yuri K. Chamorovskiy, Artem N. Fedorov, Igor’ A. Shevtsov

**Affiliations:** 1 Kotelnikov Institute of Radioengineering and Electronics of RAS, 11-7, Mokhovaya str., Moscow 125009, Russia; a.bazakutsa@optel.ru; 2 Kotelnikov Institute of Radioengineering and Electronics of RAS (Fryazino Branch), Vvedenskogo sq.1, Moscow reg, Fryazino 141120, Russia; yurichamor@fireras.su; 3 Prolog LLC, P.O. Box 3007, Kalujskiy Region, Obninsk 249033, Russia; artyf@prolog.ltd (A.N.F.); prolog@prolog.ltd (I.A.S.)

**Keywords:** optical fiber sensor, fiber Bragg gratings, bend sensor, multicore fiber

## Abstract

In this paper, we demonstrated a novel, all-fiber highly sensitive bend sensor based on a four-core fiber rod with a diameter of 2.1 mm. We observed a high resolution of the sensor at a level of 3.6 × 10^−3^ m^−1^. Such a sensor design can be used in harsh environments due to the relatively small size and all-fiber configuration, containing no adhesive, nor welded joints.

## 1. Introduction

Fiber Bragg gratings (FBGs) are widely used as selectors of optical channels, optical filters, and mirrors for fiber lasers [1]. Bragg structures are also extensively applied as sensors of physical values, such as temperature and mechanical deformation [2,3]. A typical FBG is a periodic structure with a specific period *Λ* inscribed in the core of a single-mode optical fiber. This structure reflects the light in a narrow spectral range at the Bragg wavelength *λ_B_ = 2nΛ*, where *n* is the effective refractive index for the propagating fiber’s mode. In addition to a high measurement accuracy, the key advantage of such sensors is their utility to be combined into arrays by inscribing the Bragg structures in a single optical fiber with its own Bragg wavelength for each sensor.

Currently, such sensors are widely used for the structure monitoring of bridges, buildings, complex engineering constructions and replacing electrical analogs [2]. Bragg sensors provide a high resolution of about 1 micro-strain. That is very important for a number of tasks in which it is necessary to ensure high accuracy of linear deformation measuring. This type of sensor is suitable for measuring bend deformations as well. However, to increase sensitivity, the sensor has to be installed as far as possible from the central axis of the bend. To measure bend deformations inside channels and pipes, special composite structures have to be designed, based on transducers that convert bending deformations to a linear deformation of the sensors [4,5,6,7,8,9,10] attached to the side surface of the transducer. Such sensor systems allow, for example, to conduct the measurements by inserting the sensing element into the channel of the object under investigation.

The high temperature cross-sensitivity of the Bragg sensors necessitates applying special temperature compensation sensors in the measuring setup [11,12,13]. Another solution is applying differential measurement schemes that are insensitive to temperature, but require several independent lines of sensing elements [7,14]. Most commonly, the two-axis bend deformation sensor has a circle cross-section [5,6,7]. Its diameter is determined by the application area, as well as the required sensitivity to the bending deformation magnitude. 

In some cases, it is necessary to use compact sensors of small diameters (2–10 mm), which is challenging when using combined structures. It should be noted that the accuracy and reliability of such composite systems depend upon the installation methods, materials and operating conditions. Moreover, the sensitivity of the sensor deteriorates as the diameter of the sensor decreases. At the same time, the probability of error increases due to the instability of the adhesive joints, which have non-zero plasticity. The difference in the thermal expansion coefficients of structural elements may also be a reason for the increase in the measurement errors. Application of such sensors in the nuclear industry deserve a special mention, namely for monitoring the technological parameters of nuclear reactors. For instance, it is essential to control the curvature of the reactor’s technological channels at high temperatures and increased background radiation [4]. Harsh operating conditions impose severe restrictions on the design of sensors. For example, facing high level of ionizing radiation, it is impossible to use adhesive compounds. Some design features, e.g., metal bases, makes them inapplicable, considering high temperatures and the additional radiation heating of a reactor under operation. Therefore, the development of all-fiber, radiation-resistant bending sensors featuring high sensitivity is of great interest.

A number of papers [15,16,17,18,19,20,21] have proposed bend sensors based on multicore optical fibers or fiber assemblies with Bragg gratings. The bend magnitude of those sensors is also determined by the principle of the differential scheme. However, the sensitivity of such sensors to small bends remains low, due to the small diameter of the fiber. The possible solution for increasing the sensitivity of fiber bend sensors is manufacturing combined schemes based on a Fabry-Perot interferometer [22]. The interferometer is produced by two Bragg gratings inscribed, as in previously mentioned setups, in multicore fibers. Such sensors are high-sensitive, but they have a significant disadvantage of being difficult to combine into arrays for manufacturing long quasi-distributed sensor systems. In [23], the combined bend sensor based on twin-core few-mode fiber with inscribed grating was proposed. The sensor demonstrated relatively high sensitivity. However, sensitivity of such sensors strongly depends on the bend direction angle.

In this paper, we propose an original, all-fiber design of a high-sensitive bend sensor based on Bragg structures.

## 2. Materials and Methods

**Figure 1:**
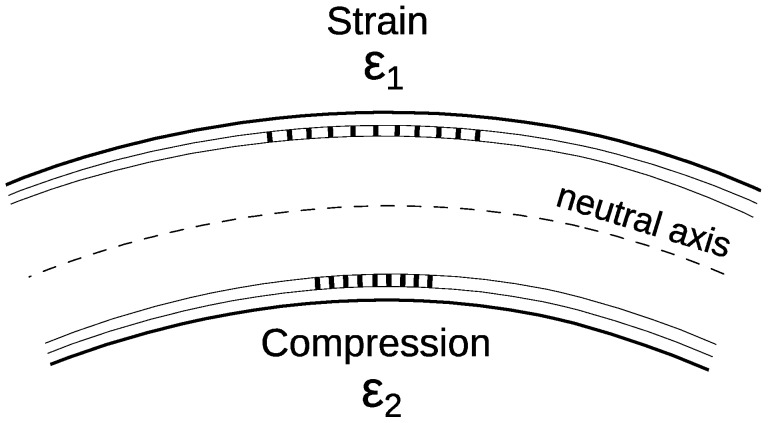
The principle of the differential scheme operation for deformation measuring.

We manufactured a few samples of the sensors for the experiments. The sensors’ design is based on the bend measurement differential principle by means of two sensitive elements located on different sides of its structure (Figure 1) [7,14]. Such an arrangement of the sensing elements allowed us to increase the measurement accuracy and get rid of the temperature influence, since the difference of the sensors’ readings rather than absolute values is used to measure the magnitude of the deformation. Measuring the magnitude of the bend in two directions forces us to use at least three sensing elements, or two pairs of them, orthogonal to each other in the cross-section of the sensor.

This measurement principle was used in combined structures of fiber bend sensors meant to control the curvature of the technological channels of an atomic reactor under repair [4], for the early warning of landslides and other geological tasks [5,6]. The principle of measurement using three- and four-core fibers is well described in literature [15,16,17,18,19,20,21]. However, the aforementioned sensors, based on multicore fibers, have a small diameter and a small distance between the cores (of about 50–70 μm). For this reason, they cannot be used as high-sensitive instruments for measuring small bend values.

The key feature of the design of the new bend sensor is a specially developed silica glass fiber rod with the external diameter of 2.1 mm. This rod contains four light-guiding, single-mode cores with a diameter of 7–8 μm. The cores are evenly spaced through 90° along the perimeter of the rod cross-section at a distance of about 50 μm from the rod surface. The structure of the rod was formed at the stage of the fiber preform fabrication. The rod was drawn with a fiber drawing tower and covered with an acrylate protective layer. To connect the rod to the spectrometric equipment, standard single-mode fibers, with 1-mm diameter ferules on the ends, were glued with immersion glue to the edge of the rod positioned correspondingly to the cores. The alignment of these fibers during the glue bonding was carried out according to the level of the output light signal. The estimated coupling losses at each joint did not exceed 2 dB. The available technology allowed us to achieve good reproducibility of the sensors manufacturing.

Bragg gratings with equal *λ_B_* were inscribed in each core in one cross-section. The gratings were inscribed by means of point-by-point inscription technology [24], but in our case, with the second harmonic radiation of a femtosecond ytterbium fiber laser. The pulse duration was ~300 fs, the energy of the pulse during recording was about 100 nJ. Applying the second harmonic of the ytterbium-doped fiber laser at 532 nm allowed us to minimize the focusing spot of the laser beam. Thus the first-order Bragg gratings inscription by the point-by-point method with high contrast is simplified.

**Figure 2:**
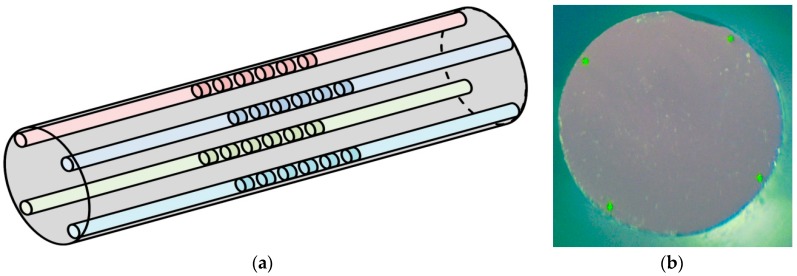
(**a**) Schematic design of a section of four-core silica rod with Bragg gratings; (**b**) Photo of the rod’s edge.

The schematic design of the rod with Bragg gratings and a picture of its end face with the cores illuminated by a green laser radiation are shown in Figure 2.

Due to the rod diameter being relatively large compared to a standard optical fiber and, as a consequence, having light-guiding cores located far from the central axis, such a sensor has a much higher sensitivity. In case of two orthogonal sensing elements, the total deformation ε during the process of the rod bending can be estimated from the formula [16]:

(1) $$\varepsilon =\varepsilon_{1}-\varepsilon_{2}=\frac{d}{R}$$

where *ε*_1_ and *ε*_2_ are the deformations of the upper and lower gratings, respectively (Figure 1), *d* is the distance between the cores, and *R* is the bend radius of the rod. Taking into account the photo-elastic coefficient of the fiber *p_e_*, the total wavelength shift of the Bragg gratings Δ*λ* can be written as:

(2) $$\Delta \lambda =\frac{\lambda_{B}\left(1-p_{e}\right)d}{2R}$$

The sensitivity of the Bragg grating with a resonant wavelength near 1.5 μm is approximately 1.1–1.2 pm/με [3]. Considering the fact that the standard resolution of the grating-assisted system is 1–2 pm, and taking into account that the distance between the cores is 2 mm, the expected minimum detectable curvature *κ* = 1/*R* can be estimated as 10^−3^ m^−1^.

## 3. Experiment and Results

**Figure 3:**
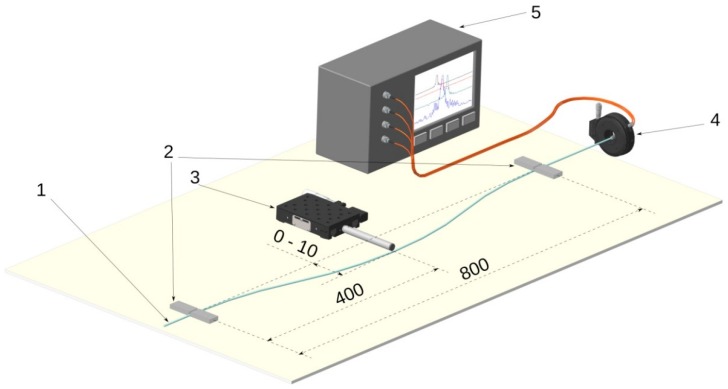
Experimental setup: 1—four core fiber, 2—side supports, 3—micrometer screw, 4—rotation mount, 5—interrogator.

The experimental setup is shown in Figure 3. The sensor was mounted on the side supports located at a distance of *L* = 800 mm between each other. The supports fixed the rod in a straight position, while also allowing its longitudinal slip to minimize possible longitudinal deformations of the sensor during its bending process. At the edge, the rod was installed in a rotation mount, which provided the rotation of the rod around its axis to perform measurements at different positions of the cores relative to the direction of the bend. The bend was performed with the help of a micrometer screw located in the center of the sensor close to the section with Bragg gratings. The spectrum was measured by means of a MicronOptics SM125-200 four-channel Bragg interrogator.

For simplicity, the shape of the rod deformation curve in such a configuration can be divided into two equal sections from the attachment point to the force application point (*L*/2), each of which is well described by the following relation [20,25]:

(3) $$\omega (x)=12\frac{a}{{\left(L/2\right)}^{2}}\left(\frac{x^{2}}{4}-\frac{x^{3}}{3L}\right)$$

where *ω*(*x*) is the deviation from the straight line along the *x* coordinate, and *a* is the magnitude of the transverse deformation at the point of the force application.

In general, the curvature κ can be calculated with the Formula (4):

(4) $$\kappa =\frac{\omega^{\prime \prime }\left(x\right)}{{\left(1+{\left(\omega^{\prime }\left(x\right)\right)}^{2}\right)}^{\frac{3}{2}}}$$

In case of small values of deformation (i.e., when the first derivative *ω’*(*x*) is small), the curvature κ can be calculated by simplifying expression (4), calculating only the second derivative of the function (3):

(5) $$\kappa =\omega^{\prime \prime }\left(x\right)=12\frac{a}{{\left(L/2\right)}^{2}}\left(\frac{1}{2}-\frac{2x}{L}\right),$$

For *L* = 800 at the point of the force application (*x* = *L*/2), expression (5) takes the following form:

(6) $$\kappa =\frac{1}{R}=-\frac{6a}{400^{2}},$$

**Figure 4:**
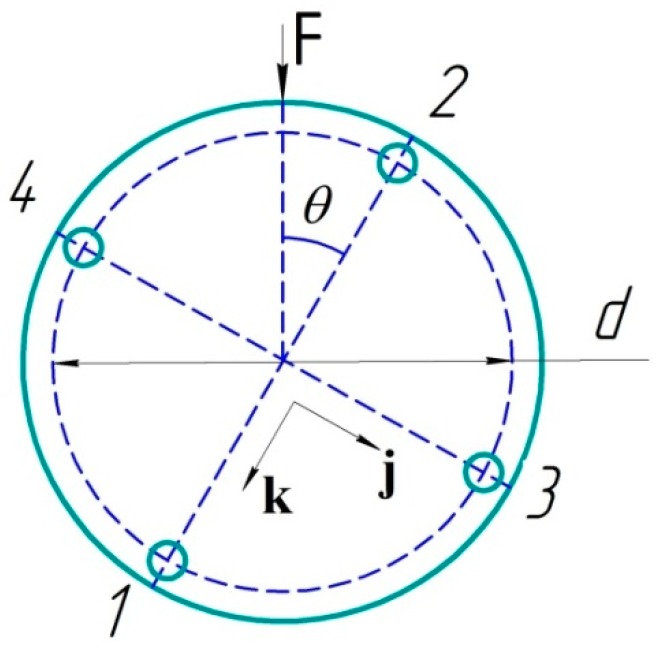
The diagram of the influence on the sensor. The angle *θ* determines the angle between the direction of external force **F** applied onto the sensor and the axis, going through the cores 2 and 1.

The cross-section of the sensor with four cores is schematically shown in Figure 4. Vector **F** indicates the direction of force applied onto the sensor. In our calculations, we used the angle *θ* between the vector **F** and the axis going through cores 2 and 1 in the sensor cross-section. The direction of this axis is determined by the unit vector **k**. The orthogonal axis going through the cores 4 and 3 is also denoted by the unit vector **j**. In our work, we conducted experiments for three different values of the angle *θ*.

**Figure 5:**
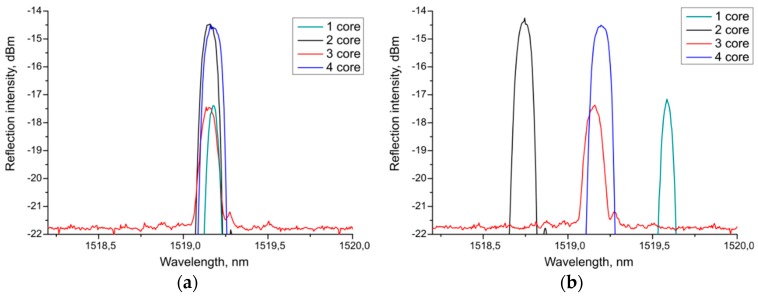
The spectra of four channels of the sensor: (**a**) Straight sensor; (**b**) under the transverse deformation *a* = 10 mm and θ ≈ −2°.

Figure 5 shows the initial spectra of the sensor for all four channels (a) and the spectra for its bend (b) with a curvature *κ* = 0.47 m^−1^ (transverse deformation *a* = 10 mm) and the angle of influence *θ* of approximately −2°. We determined the Bragg wavelength through the standard software of the interrogator without averaging the data.

**Figure 6:**
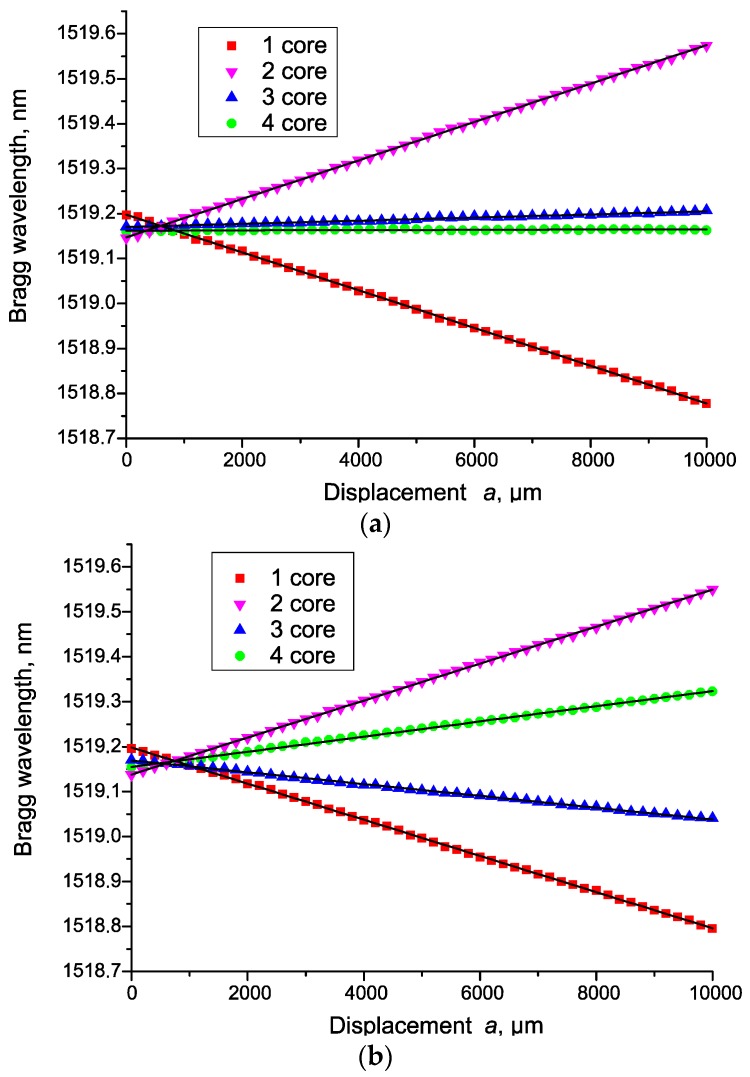

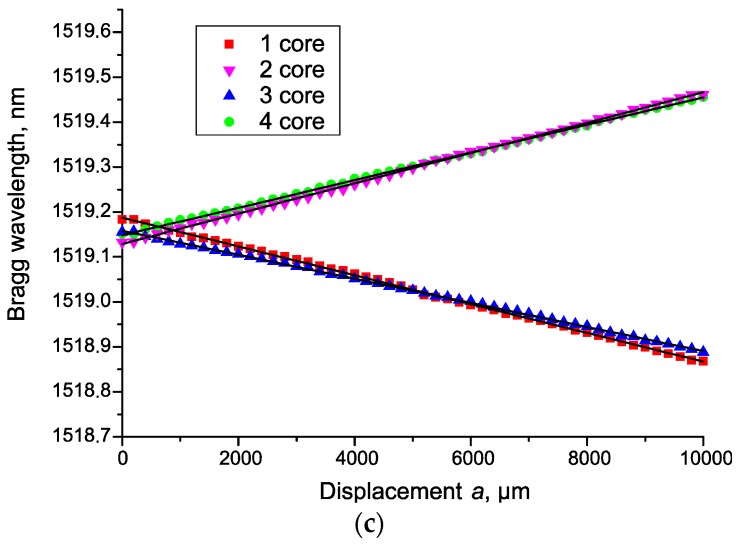
Sensor’s readings depending on the linear deformation *a* for four channels at: (**a**) *θ* ≈ −2.2°; (**b**) *θ* ≈ 20.1°; (**c**) *θ* ≈ 41.6°.

The results of the experiments are shown in Figure 6, in the form of dependences of the change in the sensors’ Bragg wavelengths on the magnitude of the linear influence *a*. At a small angle *θ*, the magnitude of the resulting deformation can be calculated by Formula (2) or (1), considering the sensitivity being equal to 1.1 pm/με. Such influence is obviously a special case of the influence along an arbitrary axis. In general, the magnitude and direction of the deformation, namely the curvature *κ* for a four-core sensor, can be calculated by the general Formula (7):

(7) $$\kappa =\frac{1}{R}=\frac{(\varepsilon_{1}-\varepsilon_{2})\cdot \cos\theta j+(\varepsilon_{3}-\varepsilon_{4})\cdot \sin\theta k}{d},$$

where *ε*_1_, *ε*_2_, *ε*_3_, and *ε*_4_ are the values of the relative deformations of the Bragg gratings located in the cores 1, 2, 3 and 4, respectively. The unit vectors **j**, **k** coincide with the axes drawn through the cores 2–1 and 4–3, respectively (Figure 4). It should be noted that we carried out the coarse measurement of the angle *θ* with the help of the rotation mount dial. We have made a more accurate calculation of the actual angle of force application, according to the results of measurements with the help of Formula (8):

(8) $$\theta =\arctan\left(\frac{\varepsilon_{3}-\varepsilon_{4}}{\varepsilon_{1}-\varepsilon_{2}}\right)$$

Obviously, at the zero values of the denominator, the angle *θ* becomes 0 or π, depending on the sign of the numerator. The caption to Figure 6 shows the values of actual angles calculated by Formula (8).

**Figure 7:**
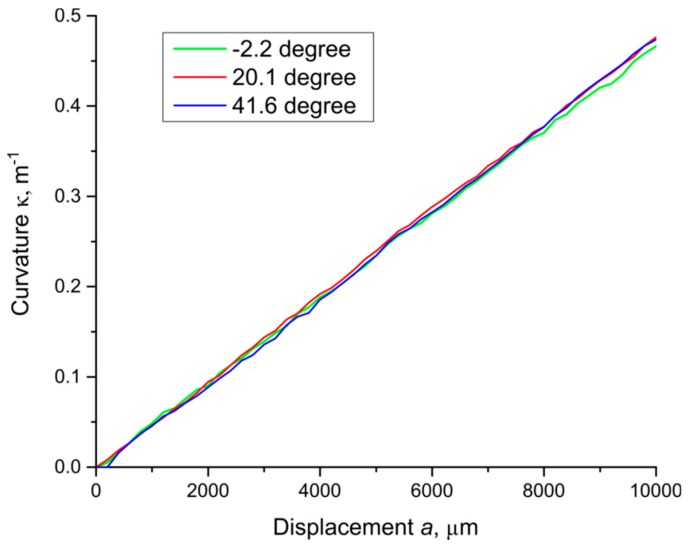
Experimentally obtained values of the curvature *κ* of the rod at the point of force application depending on the transverse deformation *a* for three different values of the angle *θ*.

Applying expression (7) to the experiment’s results, we calculated the value of the curvature *κ* at the point of force application for each experiment. The results for three different angles *θ* are presented in Figure 7. The figure below shows that the calculation of the curvature at different angles of force application *θ* does not lead to any systematic errors. As a result, the dependence of the calculated curvature on the magnitude of the linear transverse influence *a* at different angles is almost the same.

Measurement error Δ*κ* can be estimated with a well-known formula using partial derivatives:

(9) $$\Delta \kappa =\sqrt{{\sum_{n=1}^{4}\left(\frac{\partial \kappa }{\partial \varepsilon_{n}}\Delta \varepsilon_{n}\right)}^{2}},$$

where Δ*ε_n_* is the standard deviation of the measured value. Considering that this value is approximately the same (we denote it by Δ*ε*), and the angle *θ* does not change during measurement for all *ε_n_*, it can be shown that:

(10) $$\Delta \kappa =\frac{\sqrt{2}\Delta \varepsilon }{d}$$

We fit all experimental data by means of a linear function, also shown in the figures as straight lines (Figure 6). The standard deviation calculated for the series of experiments was Δ*ε* = 1.7 µε. The error in determining the curvature κ does not exceed the value Δ*κ* = 1.2 × 10^−3^ m^−1^. The triple standard deviation corresponds to the resolution of the sensor [26,27] that can be estimated as 3.6 × 10^−3^ m^−1^.

Thanks to the solid silica glass structure and the absence of adhesive joints, such a sensor retains a high accuracy and stability of readings, and opens up the prospects of being applied at high temperatures and under ionizing radiation conditions.

## 4. Conclusions

In this paper, we proposed the novel design of an all-fiber grating-assisted bend sensor, featuring Bragg gratings inscribed in four cores of a silica glass fiber rod assembly with the external diameter of 2.1 mm. The sensor’s high resolution of 3.6 × 10^−3^ m^−1^ was demonstrated. The absence of adhesive joints and components of alien materials in the operating part of the sensor allowed us to ensure the reliable operation of the sensor in harsh environments, including high temperatures and ionizing radiation.
